# Supplementary material for: A Scoping Review and Meta-Analysis of Anti-CGRP Monoclonal Antibodies: Predicting Response
Source: Pharmaceuticals (Basel). 2023 Jun 27;16(7):934. doi: 10.3390/ph16070934 (PMC10385131; doi:10.3390/ph16070934)
Supplement: Supplementary file 1 [file pharmaceuticals-16-00934-s001.zip › Supplementary Materials.pdf]

Supplementary Material S1

Embase via Ovid search strategy:

1. migraine.mp.
2. exp migraine/
3. exp calcitonin gene related peptide/
4. erenumab.mp. or exp erenumab/
5. fremanezumab.mp. or fremanezumab/
6. galcanezumab.mp. or galcanezumab/
7. eptinezumab.mp. or exp eptinezumab/
8. (calcitonin gene related peptide adj3 antibod*).mp.
9. (cgrp adj3 antibod*).mp.
10. predict*.mp.
11. (respons* or respond*).mp.
12. 1 or 2
13. 3 or 4 or 5 or 6 or 7 or 8 or 9
14. 10 or 11
15. 12 and 13 and 14

MEDLINE via PubMed Search Query:
("migraine disorders"[MeSH Terms] OR "migraine"[Text Word]) AND ("Calcitonin Gene-Related Peptide Receptor Antagonists"[MeSH Terms] OR "galcanezumab"[Supplementary Concept] OR "erenumab"[Supplementary Concept] OR "fremanezumab"[Supplementary Concept] OR "eptinezumab"[Supplementary Concept] OR "galcanezumab"[Text Word] OR "erenumab"[Text Word] OR "fremanezumab"[Text Word] OR "eptinezumab"[Text Word] OR "cgrp antibodies"[Title/Abstract:~3] OR "cgrp antibody"[Title/Abstract:~3] OR "calcitonin gene-related peptide antibodies"[Title/Abstract:~3] OR "calcitonin gene-related peptide antibody"[Title/Abstract:~3]) AND ("predict*"[Text Word] OR "respon*"[Text Word] OR "real world"[Text Word] OR "real experience"[Title/Abstract:~3] OR "real setting"[Title/Abstract:~3])
