# Supplementary material for: A Scoping Review and Meta-Analysis of Anti-CGRP Monoclonal Antibodies: Predicting Response
Source: Pharmaceuticals (Basel). 2023 Jun 27;16(7):934. doi: 10.3390/ph16070934 (PMC10385131; doi:10.3390/ph16070934)
Supplement: Supplementary file 1 [file pharmaceuticals-16-00934-s001.zip › Supplementary Tables.pdf]

Table S1 Studies that performed specific testing to examine predictive factors for response to CGRP (-R) mAbs

First author	Pub. year	Location	N	EM/CM	CGRP (-R) mAb	Inclusion/exclusion criteria	FU (mon)	Outcome	Study design	Predictive factors	50% RR
**Alpuente [1]**	2022	Barcelona Spain	43	EM (62.8%) or CM (37.2%)	Er	I: patients with EM or CM, and age-matched healthy controls<br>E: patients taking other preventive treatments, and those with medical condition that could alter saliva (smokers, chronic pain conditions, systemic disorders such as Sjogren's syndrome)	3	50% MHD	prospective observation al study	salivary CGRP	41.7%
**Nowacze-wska [2]**	2022	Poland	123	EM (29.3%) or CM (70.7%)	Er (61%), Fr (39%)	I: consecutive migraine patients receiving CGRP mAbs, migraine with or without aura, at least 4 migraine days per month, ages 18-70 years, at least 3 months of mAb treatment, TCD examination performed at baseline<br>E: patients with inadequate temporal windows, stenosis of intracranial arteries, hemodynamically significant stenosis of the ICAs, atrial fibrillation, cardiovascular disease and other severe somatic o psychiatric disorders	3	50% MMD	retrospectiv e observation al study	Maximum flow velocity in MCAs	58.5%
**Ashina [3]**	2023	Boston, USA	43	EM (55.8%) or CM (44.2%)	Ga	I: patients aged 18-65 years, prior diagnosis of migraine (with or without aura), onset of migraine 50 years or younger<br>E: pregnancy or lactation, significant psychiatric or cognitive disorder, other significant pain problem	3	50% MMD	prospective observation al cohort study	allodynia using quantitative sensory testing	55.8%
**Peng [4]**	2022	Hamburg, Germany	26	EM (57.5%) or CM (42.3%)	Ga	I: patients 18-65 years of age, at least 4 migraine days per month, failures to 5 prophylactic treatments<br>E: comorbid pain or headache disorders, contraindications to quantitative sensory testing, severe psychiatric, neurologic or somatic comorbidities	3	30% MHD	prospective cohort study	baseline heat pain threshold	

Note:

Abbreviations: Er: Erenumab, Fr: Fremanezumab, Ga: Galcanezumab, RR: responder rate, FU: follow-up, mon: months, I: included, E: excluded, MCA: middle cerebral artery, TCD: transcranial doppler, 50% RR: 50% responder rate

Table S2 Studies that examined mainly psychologic factors as predictors of response to CGRP(-R) mAbs

First author	Pub. year	Loc-ation	N	EM/CM	CGRP (-R) mAb	Inclusion criteria	FU (mon)	End-point	Study design	Predictive factors	50% RR
**Bottiroli [5]**	2021	Pavia, Italy	75	CM	Er	I: failure of at least three different pharmacological classes of preventive therapies<br>E: dementia, previous diagnosis of psychosis, mental retardation	12	50% MMD	prospective observational study	Cluster C personality disorders, anxiety disorders, number of serious stressful events	71.0%
**Lovati [6]**	2022	Milan, Italy	97	NA	Ga (33%), Fr (13%), Er (51%)	I: patients that were treated with GCRP (-R) mAbs for 6 months<br>E: not defined	6	50% MHD	(retrospective observational study)	disinhibition, depressivity, anhedonia	54.6%
**Driessen [7]**	2022	USA	1003	EM (41.5%) or CM (58.5%)	Fr	I: patients with EM or CM, aged 18 years or older, who were treated with one or more doses of Fr<br>E: pregnancy in the prior 12 months	7	Change in MHD, 50% MHD	retrospective observational study	comorbid depression and generalized anxiety disorder, MO	53.0%

Note:

Abbreviations: Er: Erenumab, Fr: Fremanezumab, Ga: Galcanezumab, RR: responder rate, FU: follow-up, mon: months, I: included, E: excluded

Table S3 Studies that examined migraine headache characteristics, migraine history, and demographic factors as predictors of response to CGRP(-R) mAbs

First author	Pub. year	Location	N	EM/CM	CGRP (-R) mAb	Inclusion/Exclusion criteria	FU (mon)	End-point	Study design	Predictive factors	50% RR
**Vernieri [8]**	2021	Italy	156	CM	Ga	I: failure of at least two preventive treatments, or other preventive treatments not possible due to comorbidities/side effects/poor compliance<br>E: not defined	3	50% MHD	prospective multicenter observational study	triptan response, unilateral pain	64.7%
**Russo [9]**	2020	Naples, Italy	70	CM	Er	I: failure of at least four or more oral preventive medication classes, or BoNTA<br>E: comorbid psychiatric disorders	6	30, 50, and 75% red. MHD	prospective observational study	disease duration	60.0%
**Iannone [10]**	2022	Florence, Italy	203	CM	Er (47.3%), Ga (36.5%), Fr (16.3%)	I: failure of three or more classes of migraine-preventive medications<br>E: not defined	6,4	50% MMD	prospective monocentric cohort study	age, MMD at baseline	56.8%
**Barbanti [11]**	2022	Italy	208	HFEM	Er (81.2%), Ga (13.5%), Fr (5.3%)	I: failure of more than three pharmacological classes of migraine preventive medications<br>E: not defined	6	50% MHD	prospective multicenter observational study	unilateral pain, unilateral autonomic symptoms	64.9%
**Barbanti [11]**	2022	Italy	656	CM	Er (71.6%), Ga (22.1%), Fr (6.3%)	I: failure of more than three pharmacological classes of migraine preventive medications<br>E: not defined	6	50% MHD	prospective multicenter observational study	unilateral pain, unilateral autonomic symptoms, allodynia, obesity	61.4%
**Frattale [12]**	2021	Abruzzo, Italy	91	HFEM (9.9%) or CM (90.1%)	Er	I: triptan users (at least one triptan for at least three migraine attacks)<br>E: not defined	6	50% MMD	real-life observational study	triptan response	63.7%

**Schoenen [13]**	2021	Belgium	156	EM (51.3%) or CM (48.7%)	Er	I: 4 or more MMD, 2 or more previous prophylactic drug treatments<br>E: myocardial infarction, stroke, TIA, unstable angina, coronary artery bypass surgery, revascularization procedures in previous 12 months, ECG abnormalities	12	50% MHD	observational cohort study	Nr. of previous prophylactic medications	51.0%
**Caronna [14]**	2021	Spain	139	CM	Er (69.1%), Ga (30.9%)	I: consecutive patients who started treatment with anti-CGRP (-R) mAbs, more than 8 migraine days, failure of at least 3 preventive medications (including BoNTA)<br>E: not defined	6	50% MHD	prospective observational study	MO(H), headache intensity	50.4%
**Raffaelli [15]**	2023	Berlin, Germany	260	EM or CM	Er (47.3%), Fr (25.5%), Ga (27.3%)	I: migraine patients receiving CGRP (-R) mAbs<br>E: treatment duration of less than 3 months, prior participation in a registration trial with CGRP (-R) mAbs, missing headache documentation	3	>75% vs. <25% red. in MHD	retrospective cohort study	typical migraine characteristics, MO(H), depression, CM	NA
**Ihara [16]**	2023	Tokyo, Japan	101	EM (55.6%) or CM (44.4%)	Ga (54%), Fr (31%), Er (13%)	I: diagnosis of migraine, including probable migraine by a headache specialist<br>E: not defined	3	50% MMD	retrospective observational study	age, Nr. of prior treatment failures, comorbid immuno-rheumatologic diseases	54.0%

**Zecca [17]**	2023	Lugano, Switzer-land	110	EM (50%) or CM (50%)	Er	I: patients aged 18 to 70 years, migraine diagnosis at least for 1 year, 8 days with migraine per month for at least 3 months, at least 2 failures or intolerability/contraindications for all 3 classes of migraine preventives<br>E: migraine onset over 50 years, history of hemiplegic migraine, primary headache disorder other than migraine, BoNTA within 4 months before inclusion, changed migraine preventive medication within 2 months before inclusion	3	50% MMD	prospective multicenter study	age at migraine onset, Nr. of prior treatment failures, MIDAS Score, genetic variants	53.6%
**Silvestro [18]**	2021	Naples, Italy	84	CM	Er	I: CM patients aged 18-65, who failed at least 4 preventive medication classes, and BoNTA<br>E: migraine patients with psychiatric comorbidities (psychosis, bipolar disorders or severe anxious or depressive symptoms)	3	30% MHD	(retrospective observational study)	MMD at baseline, pain catastrophizing, MO(H)	65.0%
**Schiano di Cola [19]**	2023	Brescia, Italy	152	HFEM (24.3%) or CM (75.7%)	Er (55.3%), Ga (32.2%), Fr (12.5%)	I: patients previously or currently in treatment with anti-CGRP or anti-CGRP-R mAbs, available 6 month follow-up<br>E: not defined	6	50% MHD	retrospective observational study	age, gender, education, marital status, triptan response, headache location	62.6%
**Lee [20]**	2023	Seoul, South Korea	238	CM	Ga	I: patients 18 years or older, with history of migraine at least 12 months<br>E: patients with a history of cluster headache or hemiplegic migraine, age >50 years at migraine onset, history of vascular disease, pregnancy or lactation	3	50% MHD	prospective observational study	continuous CM, comorbid depression, no accompanying symptoms	64.3%

**Lowe [21]**	2022	Glasgow, UK	103	CM	Er	I: patients with chronic migraine, with treatment failures to all classes of preventive medications including BoNTA<br>E: patients with uncontrolled hypertension or pregnancy	3	50% MHD	retrospective observational study	MHD at baseline, continuous CM	22.3%
**Lekont-seva [22]**	2022	Calgary, Canada	90	EM (11.1%), CM (62.2%), CM+ (27.7%)	Er	I: patients with the diagnosis of migraine, who received at least three doses of Er<br>E: not defined	3	30% MHD	retrospective (chart review) observational	EM/CM vs CM+, employment, prior preventive failures	53.3%
**Kwon [23]**	2022	Seoul, South Korea	87	EM (25.3%), or CM (74.7%)	Ga	E: patients with concomitant primary or secondary headache diagnosis	3	50% MHD	prospective observational study	MOH, Nr. of failed medications, response to BoNTA	44.8%
**Takizawa [24]**	2022	Tokyo, Japan	52	EM (48.1%), or CM (51.9%)	Ga	I: patients who have received 3 doses of Ga, fulfilling diagnostic criteria for migraine, aged >18 years<br>E: patients who discontinued Ga before completing 3 doses	3	50% MMD	retrospective observational cohort study	MOH, GAD-7	42.3%
**Vernieri [25]**	2022	Rome, Italy	191	HFEM (22.5%), or CM (77.5%)	Ga	I: consecutive patients aged 18 years or older, with a diagnosis of HFEM (8-14 MMD) or CM, with indication for Ga treatment<br>E: not defined	12	Persistent vs. nonpersistent response	prospective multicenter observational study	triptan response, gastrointestinal and psychiatric comorbidity, BMI	56.5%
**Baraldi [26]**	2021	Modena, Italy	111	CM with MOH	Er	I: patients with CM complicated with MOH, aged 18-65 years, failed at least 3 classes of first-choice preventive treatments for migraine<br>E: not defined	12	50% MHD	retrospective observational	sex, duration of medication overuse, number of previously failed preventive treatments, MHD, AC, MMD, MIDAS	44.1%

**Salem-Abdou [27]**	2021	Québec, Canada	172	EM (32%) or CM (68%)	Er	I: patients at least 18 years of age, with episodic or chronic migraine for at least 1 year, failure of at least 2 oral preventive treatments<br>E: not defined	3	50% MMD	retrospective observational	CM, TTH, triptan response	57.0%
**Torres-Ferrús [28]**	2021	Barcelona, Spain	155	EM (12.9%) or CM (87.1%)	Er (70.3%), Ga (29.7%)	I: patients with EM or CM with a headache frequency of 8 or more days per month, at least 3 preventive medication failures<br>E: not defined	3	50% MHD	prospective observational study	baseline MMD, concomitant preventive treatment, MIDAS score at baseline	39.5%
**Pensato [29]**	2022	Bologna, Italy	149	CM with MOH	Er	I: patients aged 18-65 years, migraine onset <40 years, CM with MOH, failure to BoNTA and at least 3 other preventives<br>E: pregnancy or lactation, major cardiovascular/cerebrovascular conditions, headache disorders other than CM or MOH	3	50% MHD	prospective multicentric observational cohort study	Allodynia	51.0%
**Argyriou [30]**	2023	Greece	204	HFEM (47.5%) or CM (52.5%)	Fr	I: patients with HFEM or CM, with or without MOH<br>E: contraindication to Fr, patients with major psychiatric disorder, pregnancy or lactation	3	50% MHD	prospective multicenter clinical study	MOH, presence of aura	72.5%
**Ornello [31]**	2021	Global	1410	EM (26.5%) or CM (73.5%)	Er	I: Er treatment for migraine prevention, migraine diaries, follow-up 12 weeks<br>E: not defined	3	Change in MHD	pooled patient-level analysis of observational data	sex, with additional analysis on baseline characteristics	46.5%
**Cetta [32]**	2022	Milan, Italy	30	EM (40%) or CM (60%)	Er	I: patients over and under 65 years, matched according to sex and disease activity (MHD and MMD)<br>E: not defined	6	change in MHD	prospective single-center observational study	age over 65 vs. younger	

**Guerzoni [33]**	2023	Modena, Italy	233	EM (17.2%) or CM (82.8%)	Er (72.5%), Ga (22.8%), Fr (4.7%)	I: female patients with EM or CM, with or without MO<br>E: not defined	12	change in MHD	prospective single center study	post- vs. premenopause	
**Cheng [34]**	2020	Australia	170	CM	Er	I: patients with CM, 18 years or older<br>E: pregnant or breastfeeding patients, patients with major cardio- or cerebrovascular disease, malignancy	6	50% MMD	retrospective observational	continuous CM, more than 5 previous failures to preventive medications, failure to BoNTA	46.5%
**Barbanti [35]**	2021	Italy	221	HFEM (25.8%) or CM (74.2%)	Er	I: consecutive patients aged 18-65 with HFEM or CM, with or without MO, not previously involved in any CGRP mAb RCT<br>E: not defined	12	change in MMD/ MHD	multicenter cohort study	Cutaneous allodynia, MHD at baseline, sex, prior treatment failures, psychiatric comorbidities	70.6%
**Ornello [36]**	2020	Abruzzo, Italy	89	EM (5.6%) or CM (94.4%)	Er	I: patients aged 18 to 65 years consecutively treated with erenumab	6	50% MMD	retrospective observational study	MMD at baseline, analgesic days	71.9%
**De Matteis [37]**	2022	Abruzzo, Italy	136	EM (22.8%) or CM (77.2%)	Er, Ga, Fr	I: consecutive patients treated with CGRP (-R) mAbs	3	change in MHD	prospective observational study	cranial autonomic symptoms	56.8%
**De Vries Lentsch [38]**	2022	Netherlands	94	EM (55%) or CM (45%)	Er	I: migraine patients (episodic or chronic, with or without aura) that started erenumab<br>E: comorbid primary headache disorder other than tension type headache	3	change in MMD	prospective observational study	serum CGRP-like immunoreactivity	

Note:

Abbreviations: Er: Erenumab, Fr: Fremanezumab, Ga: Galcanezumab, Ept: Eptinezumab RR: responder rate, FU: follow-up, mon: months, I: included, E: excluded, BoNTA: onabotulinumtoxinA

List of included studies

1. Alpuente, A.; Gallardo, V.J.; Asskour, L.; Caronna, E.; Torres-Ferrus, M.; Pozo-Rosich, P. Salivary CGRP and Erenumab Treatment Response: Towards Precision Medicine in Migraine. *Annals of Neurology* **2022**, *92*, 846–859, doi:10.1002/ana.26472.
2. Nowaczewska, M.; Straburzyński, M.; Waliszewska-Prosół, M.; Meder, G.; Janiak-Kiszka, J.; Kaźmierczak, W. Cerebral Blood Flow and Other Predictors of Responsiveness to Erenumab and Fremanezumab in Migraine—A Real-Life Study. *Front. Neurol.* **2022**, *13*, 895476, doi:10.3389/fneur.2022.895476.
3. Ashina, S.; Melo-Carrillo, A.; Szabo, E.; Borsook, D.; Burstein, R. Pre-Treatment Non-Ictal Cephalic Allodynia Identifies Responders to Prophylactic Treatment of Chronic and Episodic Migraine Patients with Galcanezumab: A Prospective Quantitative Sensory Testing Study (NCT04271202). *Cephalalgia* **2023**, *43*, 033310242211478, doi:10.1177/03331024221147881.
4. Peng, K.-P.; Basedau, H.; Oppermann, T.; May, A. Trigeminal Sensory Modulatory Effects of Galcanezumab and Clinical Response Prediction. *Pain* **2022**, *163*, 2194–2199, doi:10.1097/j.pain.0000000000002614.
5. Bottiroli, S.; De Icco, R.; Vaghi, G.; Pazzi, S.; Guaschino, E.; Allena, M.; Ghiotto, N.; Martinelli, D.; Tassorelli, C.; Sances, G. Psychological Predictors of Negative Treatment Outcome with Erenumab in Chronic Migraine: Data from an Open Label Long-Term Prospective Study. *J Headache Pain* **2021**, *22*, 114, doi:10.1186/s10194-021-01333-4.
6. Lovati, C.; Bernasconi, G.; Capogrosso, C.; Molteni, L.; Giorgetti, F.; Dell'Osso, B.; Pantoni, L. Personality Traits and Efficacy of Anti-CGRP Monoclonal Antibodies in Migraine Prevention. *Neurol Sci* **2022**, *43*, 5765–5767, doi:10.1007/s10072-022-06251-0.
7. Driessen, M.T.; Cohen, J.M.; Patterson-Lomba, O.; Thompson, S.F.; Seminerio, M.; Carr, K.; Totev, T.I.; Sun, R.; Yim, E.; Mu, F.; et al. Real-World Effectiveness of Fremanezumab in Migraine Patients Initiating Treatment in the United States: Results from a Retrospective Chart Study. *J Headache Pain* **2022**, *23*, 47, doi:10.1186/s10194-022-01411-1.
8. Vernieri, F.; Altamura, C.; Brunelli, N.; Costa, C.M.; Aurilia, C.; Egeo, G.; Fofi, L.; Favoni, V.; Lovati, C.; Bertuzzo, D.; et al. Rapid Response to Galcanezumab and Predictive Factors in Chronic Migraine Patients: A 3-month Observational, Longitudinal, Cohort, Multicenter, Italian Real-life Study. *Euro J of Neurology* **2022**, *29*, 1198–1208, doi:10.1111/ene.15197.
9. Russo, A.; Silvestro, M.; Scotto di Clemente, F.; Trojsi, F.; Bisecco, A.; Bonavita, S.; Tessitore, A.; Tedeschi, G. Multidimensional Assessment of the Effects of Erenumab in Chronic Migraine Patients with Previous Unsuccessful Preventive Treatments: A Comprehensive Real-World Experience. *J Headache Pain* **2020**, *21*, 69, doi:10.1186/s10194-020-01143-0.
10. Iannone, L.F.; Fattori, D.; Benemei, S.; Chiarugi, A.; Geppetti, P.; De Cesaris, F. Long-Term Effectiveness of Three Anti-CGRP Monoclonal Antibodies in Resistant Chronic Migraine Patients Based on the MIDAS Score. *CNS Drugs* **2022**, *36*, 191–202, doi:10.1007/s40263-021-00893-y.
11. Barbanti, P.; Egeo, G.; Aurilia, C.; Altamura, C.; d'Onofrio, F.; Finocchi, C.; Albanese, M.; Aguggia, M.; Rao, R.; Zucco, M.; et al. Predictors of Response to Anti-CGRP Monoclonal Antibodies: A 24-Week, Multicenter, Prospective Study on 864 Migraine Patients. *J Headache Pain* **2022**, *23*, 138, doi:10.1186/s10194-022-01498-6.
12. Frattale, I.; Caponnetto, V.; Casalena, A.; Assetta, M.; Maddestra, M.; Marzoli, F.; Affaitati, G.; Giamberardino, M.A.; Viola, S.; Gabriele, A.; et al. Association between Response to Triptans and Response to Erenumab: Real-Life Data. *J Headache Pain* **2021**, *22*, 1, doi:10.1186/s10194-020-01213-3.
13. Schoenen, J.; Timmermans, G.; Nonis, R.; Manise, M.; Fumal, A.; Gérard, P. Erenumab for Migraine Prevention in a 1-Year Compassionate Use Program: Efficacy, Tolerability, and Differences Between Clinical Phenotypes. *Front. Neurol.* **2021**, *12*, 805334, doi:10.3389/fneur.2021.805334.
14. Caronna, E.; Gallardo, V.J.; Alpuente, A.; Torres-Ferrus, M.; Pozo-Rosich, P. Anti-CGRP Monoclonal Antibodies in Chronic Migraine with Medication Overuse: Real-Life Effectiveness and Predictors of Response at 6 Months. *J Headache Pain* **2021**, *22*, 120, doi:10.1186/s10194-021-01328-1.
15. Raffaelli, B.; Fitzek, M.; Overeem, L.H.; Storch, E.; Terhart, M.; Reuter, U. Clinical Evaluation of Super-Responders vs. Non-Responders to CGRP(-Receptor) Monoclonal Antibodies: A Real-World Experience. *J Headache Pain* **2023**, *24*, 16, doi:10.1186/s10194-023-01552-x.
16. Ihara, K.; Ohtani, S.; Watanabe, N.; Takahashi, N.; Miyazaki, N.; Ishizuchi, K.; Hori, S.; Takemura, R.; Nakahara, J.; Takizawa, T. Predicting Response to CGRP-Monoclonal Antibodies in Patients with Migraine in Japan: A Single-Centre Retrospective Observational Study. *J Headache Pain* **2023**, *24*, 23, doi:10.1186/s10194-023-01556-7.

17. Zecca, C.; Cargnin, S.; Schankin, C.; Giannantoni, N.M.; Viana, M.; Maraffi, I.; Riccitelli, G.C.; Sihabdeen, S.; Terrazzino, S.; Gobbi, C. Clinic and Genetic Predictors in Response to Erenumab. *Euro J of Neurology* **2022**, *29*, 1209–1217, doi:10.1111/ene.15236.
18. Silvestro, M.; Tessitore, A.; Scotto di Clemente, F.; Battista, G.; Tedeschi, G.; Russo, A. Refractory Migraine Profile in CGRP-monoclonal Antibodies Scenario. *Acta Neuro Scandinavica* **2021**, *144*, 325–333, doi:10.1111/ane.13472.
19. Schiano di Cola, F.; Bolchini, M.; Ceccardi, G.; Caratozzolo, S.; Liberini, P.; Rao, R.; Padovani, A. An Observational Study on Monoclonal Antibodies against Calcitonin-gene-related Peptide and Its Receptor. *Euro J of Neurology* **2023**, ene.15761, doi:10.1111/ene.15761.
20. Lee, H.C.; Cho, S.; Kim, B.-K. Predictors of Response to Galcanezumab in Patients with Chronic Migraine: A Real-World Prospective Observational Study. *Neurol Sci* **2023**, doi:10.1007/s10072-023-06683-2.
21. Lowe, M.; Murray, L.; Tyagi, A.; Gorrie, G.; Miller, S.; Dani, K.; the NHS Greater Glasgow and Clyde Headache Service Efficacy of Erenumab and Factors Predicting Response after 3 Months in Treatment Resistant Chronic Migraine: A Clinical Service Evaluation. *J Headache Pain* **2022**, *23*, 86, doi:10.1186/s10194-022-01456-2.
22. Lekontseva, O.; Wang, M.; Amoozegar, F. Predictors of Clinical Response to Erenumab in Patients with Migraine. *Cephalalgia Reports* **2022**, *5*, 251581632211281, doi:10.1177/25158163221128185.
23. Kwon, S.; Gil, Y.-E.; Lee, M.J. Real-World Efficacy of Galcanezumab for the Treatment of Migraine in Korean Patients. *Cephalalgia* **2022**, *42*, 705–714, doi:10.1177/03331024221076481.
24. Takizawa, T.; Ohtani, S.; Watanabe, N.; Miyazaki, N.; Ishizuchi, K.; Sekiguchi, K.; Iba, C.; Shibata, M.; Takemura, R.; Hori, S.; et al. Real-World Evidence of Galcanezumab for Migraine Treatment in Japan: A Retrospective Analysis. *BMC Neurol* **2022**, *22*, 512, doi:10.1186/s12883-022-03041-1.
25. Vernieri, F.; Brunelli, N.; Marcosano, M.; Aurilia, C.; Egeo, G.; Lovati, C.; Favoni, V.; Perrotta, A.; Maestrini, I.; Rao, R.; et al. Maintenance of Response and Predictive Factors of 1-year GALCANEZUMAB Treatment in Real-life Migraine Patients in ITALY : The Multicenter Prospective Cohort GARLIT Study. *Euro J of Neurology* **2023**, *30*, 224–234, doi:10.1111/ene.15563.
26. Baraldi, C.; Castro, F.L.; Cainazzo, M.M.; Pani, L.; Guerzoni, S. Predictors of Response to Erenumab after 12 Months of Treatment. *Brain and Behavior* **2021**, *11*, doi:10.1002/brb3.2260.
27. Salem-Abdou, H.; Simonyan, D.; Puymirat, J. Identification of Predictors of Response to Erenumab in a Cohort of Patients with Migraine. *Cephalalgia Reports* **2021**, *4*, 251581632110266, doi:10.1177/25158163211026646.
28. Torres-Ferrús, M.; Gallardo, V.J.; Alpuente, A.; Caronna, E.; Gine-Cipres, E.; Pozo-Rosich, P. The Impact of Anti-CGRP Monoclonal Antibodies in Resistant Migraine Patients: A Real-World Evidence Observational Study. *J Neurol* **2021**, *268*, 3789–3798, doi:10.1007/s00415-021-10523-8.
29. Pensato, U.; Baraldi, C.; Favoni, V.; Cainazzo, M.M.; Torelli, P.; Querzani, P.; Pascazio, A.; Mascarella, D.; Matteo, E.; Quintana, S.; et al. Real-Life Assessment of Erenumab in Refractory Chronic Migraine with Medication Overuse Headache. *Neurol Sci* **2022**, *43*, 1273–1280, doi:10.1007/s10072-021-05426-5.
30. Argyriou, A.A.; Dermitzakis, E.V.; Xiromerisiou, G.; Rallis, D.; Soldatos, P.; Litsardopoulos, P.; Vikelis, M. Efficacy and Safety of Fremanezumab for Migraine Prophylaxis in Patients with at Least Three Previous Preventive Failures: Prospective, Multicenter, Real-world Data from a Greek Registry. *Euro J of Neurology* **2023**, *30*, 1435–1442, doi:10.1111/ene.15740.
31. Ornello, R.; Baraldi, C.; Guerzoni, S.; Lambru, G.; Fuccaro, M.; Raffaelli, B.; Gendolla, A.; Barbanti, P.; Aurilia, C.; Cevoli, S.; et al. Gender Differences in 3-Month Outcomes of Erenumab Treatment—Study on Efficacy and Safety of Treatment With Erenumab in Men. *Front. Neurol.* **2021**, *12*, 774341, doi:10.3389/fneur.2021.774341.
32. Cetta, I.; Messina, R.; Zanandrea, L.; Colombo, B.; Filippi, M. Comparison of Efficacy and Safety of Erenumab between over and under 65-Year-Old Refractory Migraine Patients: A Pivotal Study. *Neurol Sci* **2022**, *43*, 5769–5771, doi:10.1007/s10072-022-06190-w.
33. Guerzoni, S.; Baraldi, C.; Brovia, D.; Cainazzo, M.M.; Lo Castro, F.; Pani, L. Monoclonal Anti-CGRP Antibodies in Post-Menopausal Women: A Real-Life Study. *Acta Neurol Belg* **2023**, doi:10.1007/s13760-023-02190-5.
34. Cheng, S.; Jenkins, B.; Limberg, N.; Hutton, E. Erenumab in Chronic Migraine: An Australian Experience. *Headache: The Journal of Head and Face Pain* **2020**, *60*, 2555–2562, doi:10.1111/head.13968.

35. Barbanti, P.; Aurilia, C.; Cevoli, S.; Egeo, G.; Fofi, L.; Messina, R.; Salerno, A.; Torelli, P.; Albanese, M.; Carnevale, A.; et al. Long-term (48 Weeks) Effectiveness, Safety, and Tolerability of Erenumab in the Prevention of High-frequency Episodic and Chronic Migraine in a Real World: Results of the EARLY 2 Study. *Headache* **2021**, *61*, 1351–1363, doi:10.1111/head.14194.
36. Ornello, R.; Casalena, A.; Frattale, I.; Gabriele, A.; Affaitati, G.; Giamberardino, M.A.; Assetta, M.; Maddestra, M.; Marzoli, F.; Viola, S.; et al. Real-Life Data on the Efficacy and Safety of Erenumab in the Abruzzo Region, Central Italy. *J Headache Pain* **2020**, *21*, 32, doi:10.1186/s10194-020-01102-9.
37. De Matteis, E.; Caponnetto, V.; Casalena, A.; Frattale, I.; Gabriele, A.; Affaitati, G.; Giamberardino, M.A.; Maddestra, M.; Viola, S.; Pistoia, F.; et al. Cranial Autonomic Symptoms and Response to Monoclonal Antibodies Targeting the Calcitonin Gene-Related Peptide Pathway: A Real-World Study. *Front. Neurol.* **2022**, *13*, 973226, doi:10.3389/fneur.2022.973226.
38. De Vries Lentsch, S.; Garrelds, I.M.; Danser, A.H.J.; Terwindt, G.M.; MaassenVanDenBrink, A. Serum CGRP in Migraine Patients Using Erenumab as Preventive Treatment. *J Headache Pain* **2022**, *23*, 120, doi:10.1186/s10194-022-01483-z.
